# Up-regulation of microRNA miR-101-3p enhances sensitivity to cisplatin via regulation of small interfering RNA (siRNA) Anti-human AGT4D and autophagy in non-small-cell lung carcinoma (NSCLC)

**DOI:** 10.1080/21655979.2021.1982274

**Published:** 2021-10-25

**Authors:** Dong Cui, Yu Feng, Rulin Qian

**Affiliations:** Department of Thoracic Surgery, Henan Provincial Chest Hospital, Zhengzhou, China

**Keywords:** miR-101-3p, cisplatin sensitivity, autophagy, NSCLC

## Abstract

The emergence of drug resistance hinders the treatment of malignant tumors, and autophagy plays an important role in tumor chemotherapy resistance. However, its mechanism in non-small cell lung cancer (NSCLC) has not been well-researched. We aim to investigate the role of miR-101-3p in cisplatin-resistant via regulation of autophagy-related protein 4D (ATG4D) and autophagy. Cell viability, apoptosis, fluorescence intensity of GFP-LC3 and RFP-GFP-LC3 were determined using Cell Counting Kit-8 (CCK-8) assay, flow cytometry, and Laser scanning confocal microscope analysis, respectively. The levels of LC3II/LC3I, P62 and ATG4D were detected by Western blot. The results showed that the sensitivity to cisplatin in NSCLC cells was up-regulated by miR-101-3p mimics treatment, inducing promoting cell apoptosis and inhibiting autophagy. Further mechanistic study identified that ATG4D was a direct target of miR-101-3p. Moreover, ATG4D siRNA also could reverse miR-101-3p inhibitor-induced the up-regulation of ATG4D and the ration of LC3II/LC3I, the down-regulation of p62 expression. Our findings indicated that miR-101-3p could regulate sensitivity to cisplatin of NSNCC cells by regulating autophagy mediated by ATG4D. Therefore, miR-101-3p may act as a potential therapeutic target for the treatment of NSCLC.

## Introduction

Lung cancer, as the most common malignant cancer, was associated with high mortality rate all over the world, approximately 80–85% of total lung cancer cases is non-small cell-lung cancer (NSCLC) [1]. Cisplatin is widely used as the first-line chemotherapy drug for treatment in hepatocellular carcinoma (HCC), triple-negative breast cancer, bladder cancer and ovarian, also including NSCLC [2–6]. Cisplatin binding could result in DNA damage and induce cell apoptosis. However, cisplatin chemotherapy is often limited by inherent and acquired drug resistance. Therefore, it is urgent for us to search effective targets to improve treatment in NSCLC patients.

MicroRNAs (miRNAs), a class of small endogenous conserved RNAs, modulate a different kind of gene expression by interacting with their mRNAs, leading to mRNA degradation or inhibition of protein translation [7]. Accumulating evidence indicated that miRNAs are participate in various biological processes in cancer cells, including cell proliferation, apoptosis, invasion and migration [8,9]. Moreover, up-regulation or down-regulation miRNAs could help regulate cancer cell drug resistance, including NSCLC [10,11]. MiR-126-3p mimics could increase melanoma's sensitivity to dabrafenib by regulating disintegrin and metalloproteinase 9 (ADAM9) [12]. Inhibition of miR-214 could reverse resistance to erlotinib in NSCLC via increasing LIM Homeobox 6 (LHX6) [13]. The level of miR-101-3p was reduced in many cancers including NSCLC, indicating that it may act as a tumor suppressor [14–16]. Up-regulation of miR-101-3p enhanced sensitivity to oxaliplatin in HCC cells by inhibiting autophagy and Beclin-1^14^. Transfection of miR-101-3p mimics also sensitized bladder urothelial carcinoma cells to cisplatin via inhibiting enhancer of zeste homolog 2 (EZH2) expression[17]. It has been demonstrated that miR-101-3p could regulate cell autophagy in various cells, and it may be a potential autophagy inhibitor [14,18]. However, the potential mechanisms of miR-101-3p regulating drug resistance in NSCLC have not been explored. Therefore, we need to elucidate the effect of miR-101-3p on cisplatin-resistant NSCLC.

Autophagy is a highly conserved catabolic process that involves cells self-digesting through a double-membrane organelle called autophagosome [19,20]. Autophagy is composed of a series of molecular events that lead to the formation of autophagosomes, the autophagosomes engulf the intracellular material and finally fuse with lysosomes to degrade its contents [21]. It has been reported that autophagy can promote tumor progression and resistance to treatment in cancers. Over-expression of miRNA-22 could inhibit cisplatin resistance by regulation of autophagy and apoptosis in osteosarcoma [22]. Transfection of miRNA-1301 could inhibit cisplatin-resistance in human ovarian cancer cells by regulation of epithelial-to-mesenchymal transition (EMT) and NF-κB signaling pathway [23]. This study investigated the effect of miR-101a-3p alterations in drug-resistant NSCLC cells and the relationship between cisplatin resistance, miR-101-3p and autophagy. However, whether autophagy has a cytoprotective effect and how it influences the acquired resistance to cisplatin through specific molecular mechanisms was unclear. Therefore, the present study revealed that miR-101-3p may be a potential target, which can be used to counteract the drug resistance and improve treatment in NSCLC patients.

## Materials and methods

### Cell culture and cell viability

The human NSCLC cells and the normal lung epithelial cells needed for this study include (A549 PC-9, NCI-H1299, HCC827(ATCC) and MCR5 were purchased from National Collection of Authenticated Cell Cultures (Shanghai, China). All cells were maintained in PRMI 1640medium (GIBCO) contained with 10% FBS (GIBCO) in the incubator with a special environment (5% CO_2_, 37°C). When the cells were grown to a certain extent, we digested the cells, inoculated the cells in a 96-well plate at a density of 5000/well, cultured for 24 hours until the cells adhered to the wall of culture flask, and then added different concentrations of drugs to incubate for 48 hours. At last, we added 10-µl Cell Counting Kit-8 (CCK-8; Dojindo) solution that was from CCK-8 assay to detect cell viability for incubation 3 hours and then measured the OD value using a microtiter reader at 450 nm.

### Transfection

MiR-101-3p mimics, inhibitors or negative controls were designed from RiboBio Co., Ltd Company (Guangzhou, China). AGT4D siRNA and Negative control was purchased from Santa Cruz. According to the instruction, lipofectamine 2000 (Invitrogen, USA) was used for transfection. The relative sequence is shown in Table 1.

**Table 1.** The primer sequences are as follows

Name	Sequence
NC siRNA	5′- ATCCCCGAACGUGACACGUAT −3′
AGT4D siRNA NC mimics NC inhibitor	5′- CGGACCAGCUUUAGCAAGA −3′ 5ʹ- CAGUACUUUUGUGUAGUACAA −3ʹ 5ʹ- UUCUCCGAACGUGUCACGUTT −3ʹ
miR-101-3p mimics	5′- UACAGUACUGUGAUAACUGA −3′
miR-101-3p inhibitor	5′- UCAGUUAUCACAGUACUGUA −3′

### Cell proliferation

The cells were cultivated into 96-well plates and examined by a Click-iTEdU Imaging kit (Invitrogen; Thermo Fisher Scientific, Inc.) according to the manufacturer’s protocol. NSCLC cells were incubated with the IC50 concentration of Cisplatin for 24 h at 37°C, fixed by 4% paraformaldehyde at room temperature for 20 min, permeabilized by 0.5% Triton-X100 at room temperature for 15–20 min, then followed by 10 μM EdU for 2 h at room temperature for 20 min. Cell nuclei were stained by 5 μg/ml Hoechst 33,342 (Invitrogen; Thermo Fisher Scientific, Inc.) for 30 min at room temperature.

### Flow cytometry analysis

The number of apoptotic cells was determined by an Annexin V‑FITC/propidium iodide (PI) staining kit (BCD Biosciences, San Jose, CA, USA). In brief, following transfection at 48 h, the cells were cultivated into 6-well plates, and stained with 5 μL Annexin V-FITC and 10 μL PI for 15 min at 4°C in the dark. Finally, the cells were analyzed using a FACScan flow cytometer (Becton‑Dickinson, Bedford, MA, USA).

### Dual luciferase reporter assay

The wild type (WT) 3ʹ-UTR and mutated (Mut) 3ʹ-UTR of the ATG4D gene containing the predicted target sites for miR-101a-3p were constructed by GenePharma. The NSCLC cell were seeded into 24-well plates and co-transfected with 50 ng recombinant luciferase vectors, 5 ng pRL-TK vectors and 50 nM miR-101-3p mimics or inhibitor, and miR negative control (miR-NC) using Lipofectamine 2000. After transfection for 48 h, the relative luciferase activities were measured using the Dual Luciferase Reporter Assay System (Promega).

### Western blot analysis

The NSCLC cells were collected and lysed with ice-cold lysis buffer (20 mM Tris(pH7.5),150 mM NaCl, 1% Triton X-100, sodium pyrophosphate, β-glycerophosphate, EDTA, Na3VO4, leupeptin) (Beyotime Institute of Biotechnology) [24]. The protein concentrations were determined using BCA protein assay kits (Beyotime). 40 μg of protein samples were separated by 10% SDS-PAGE and then transferred onto PVDF membranes (Thermo, USA). The membranes were incubated with LC3, p62 (1:2,000; Abcam, USA) and GAPDH (1:1,000; CST; USA) overnight at 4°C, then incubated with a horseradish peroxidase-conjugated secondary antibody (1;2000; CST; USA) for 2 h at 37 °C. Finally, they were determined using ECL kits (Beyotime) and analyzed using software ImageJ v1.8.0 (National Institutes of Health).

### RNA extraction and qRT-PCR

We used TRIzol reagent to extract total RNA from cultured HCC cells; then, total RNA was reverse transcribed into cDNA by Moloney murine leukemia virus (M-MLV) reverse transcriptase (TAKARA, Dalian, China) according to the manufacturer s instructions. We used SYBR® Premix Ex Taq™ II (Takara) to analyze qRT-PCR. The 2^−ΔΔCt^ method was used to calculate the relative gene expression normalized by GAPDH and U6. The relative sequences of primers are listed in Table 2.

**Table 2.** The primer sequences are as follows

Name	Sequence
NC control	forward 5′- UUCUCCGAACGUGUCACGUTT −3′; reverse 5′- ACGUGACACGUUCGGAGAATT −3′;
miR-101-3p	forward 5ʹ- GCGGCGGTCAAGAGCAATAACG −3ʹ reverse 5ʹ- ATCCAGTGCAGGGTCCGAGG −3ʹ;
AGT4D	forward: 5ʹ- AGCTCCTCCTCAGCCACA −3 reverse: 5ʹ- GGAGCAGAGGTCGTCCAG −3’

### Laser scanning confocal microscope

Cells were transfected with pSELECT-GFP-LC3 (InvivoGen, San Diego, CA) using Lipofectamine reagent (Invitrogen) following the manufacturer’s instructions. Briefly, cells stably transfected with monomeric red fluorescent protein (mRFP)–green fluorescent protein (GFP)–LC3 adenovirus were subjected to different treatments. After 48 h, the cells were fixed with 4% paraformaldehyde (Sigma, USA). Laser confocal fluorescence microscopy was used to observe the green (GFP) and red (mRFP) fluorescence. Autophagosomes appeared as yellow dots and autolysosomes appear as red dots in merged images. The autophagic flux was determined by the increase in the percentage of red dots in the merged images.

### Electron microscopy detection

Cells were harvested and fixed in 2.5% glutaraldehyde, and then post-fixed in 1% osmium tetroxide buffer, dehydration with ethanol, embedded using epoxy resin, stained with 3% uranyl acetate and lead citrate, and examined by transmission electron microscope. Autophagic vacuoles per cell were examined by counting 10 cells for each sample.

### Nude mouse xenograft model

Twenty BALB/c nude mice (5 weeks,18–20 g old) were obtained from Charles River (Beijing, China). The mice were adaptively fed for 1 week, light/night cycle for 12 hours, indoor temperature control at 23 ± 2°C, humidity 55 ± 5%, and free drinking water. 2 × 10^7^ A549 were injected subcutaneously into the left lower limb of nude mice, and the tumor reached around 50 − 100 mm^3^, the mice were randomly divided into 3 groups with 5 animals in each group (blank control group, cisplatin treatment group, miR-101-3p agomiR+ cisplatin group). In this study, we used miR-101-3p agomiR (RiboBio, Guangzhou, China) to modify miR-101-3p mimics in vivo studies. The cisplatin (2.0 mg/kg), miR-101-3p agomiR (10 nmol), was injected intraperitoneally every 3 days for 3 weeks. We measured the tumor volume every two days and drawn the tumor growth curve. On the 5th and last day, nude mice were photographed in vivo. The tumor volume was recorded every 2 days (Tumor volume (mm^3^) = length × width [2]/2).

### TUNEL analysis

The tissue sections were fixed in 4% paraformaldehyde for 24 h, infiltrated with wax and embedded with paraffin. Then, add 100 μL TUNEL reaction mixture to the specimen and add cover glass or parafilm to react at 37°C for 1 h in a dark humidified box; wash with PBS for three times, add 100 μL streptavidin-labeled HRP (diluted in 1:500 PBS) for 30 minutes; wash it again, add 100 μL diaminobenzidine (DAB) mixture for 10 minutes, rinse with deionized water for 5 times; counterstain with hematoxylin, rinsed with tap water after about 3 seconds; dehydrate with gradient alcohol, transparent xylene for 1 minute, seale with neutral glue. Observe and take pictures under an immunofluorescence microscope (Olympus).

### Immunocytochemistry

The sections are routinely deparaffinized in water, dipped with xylene for 10 min; immersed with gradient ethanol from high concentration to low concentration for 5 min each time; then fixed with 4% paraformaldehyde for 15 min; Place the slices in Hydrogen Peroxide Block and incubate for 10 minutes, PBS immersion for 5 min, repeat 2 times, add Ultra V Block dropwise, incubated at room temperature for 5 minutes to block nonspecific background staining, wash with PBS for 5 minutes, and repeat twice. Drop the primary antibody working solution and incubate at 37°C for 2 hours; drop the Primary Antibody Enhancer and incubate at room temperature for 20 minutes, add the enzyme-labeled secondary antibody dropwise, and incubate at room temperature for 30 minutes in the dark. Finally, add 1–2 drops of DAB incubate for 10 minutes. Finally, hematoxylin was used as a counterstain, the slices were rinsed thoroughly with tap water, counterstained, dehydrated, transparent, mounted and observed under light microscopy (Olympus).

### Statistical analysis

The experimental data were presented as the mean ± standard deviation (SD) and the data were analyzed using GraphPad Prism 8.0 (GraphPad Software, Inc.). The differences between two groups or more than two groups were compared using an unpaired Student’s t-test or one-way ANOVA, respectively. The results were considered statistically significant at P < 0.05. All experiments were repeated three times.

## Results

### Screening of the key miRNA was down-regulated in LUAD tissues and NSCLC cells

**Figure 1.**
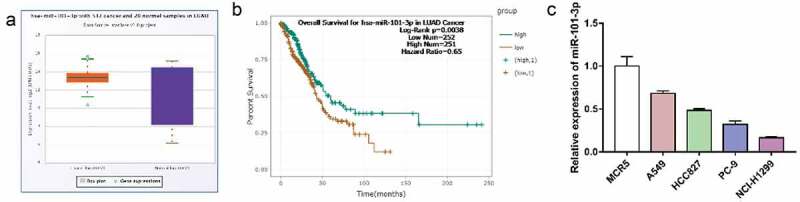
Screening of the key miRNA was down-regulated in LUAN tissues and NSCLC cells

To determine different expressions of miRNA in NSCLC, we analyzed a microarray from Gene database (number: GSE135918) to compare the level of miRNA in five paired primary LUAD tissue and adjacent nontumor tissues (ANT). We screened 15 differentially expressed miRNAs(miR-20a-5p, miR-338-3p, miR-33a-5p, miR-126-5p, miR-126-3p, miR-130a-3p, miR-199b-5p, miR-140-5p, miR-20b-5p, miR-101-3p, miR-195-5p, miR-30a-3p, miR-451a and miR-660-5p, miR-15a-5p) which were down-regulated in tumor tissues. Next, we combined with starbase3 database analysis and found that only miR-101-3p was low expressed in LUAD tissue compared with the ANT tissue and has a significant difference in survival curve (Figure 1 A-B). Furthermore, we examined miR-101-3p expression in NSCLC (A549 PC-9, NCI-H1299, HCC827(ATCC) and lung epithelial cell MCR5, resulted that it was highest in MCR5 and we chose A549 and NCI-H1299 to do the further research (Figure 1b). The above results indicate that miR-101-3p may be a tumor suppressor in NSCLC cells.

**A**. Starbase3 database analysis miR-101-3p expression in LUAD tissue compared with the ANT tissue. **B**. The Survival Analysis of miR-101-3p Genes in LUAD. **C**. The level of miR-101-3p was determined by qRT-PCR.

### MiR-101-3p mimics increased sensitivity to cisplatin and promoted cell apoptosis

**Figure 2.**
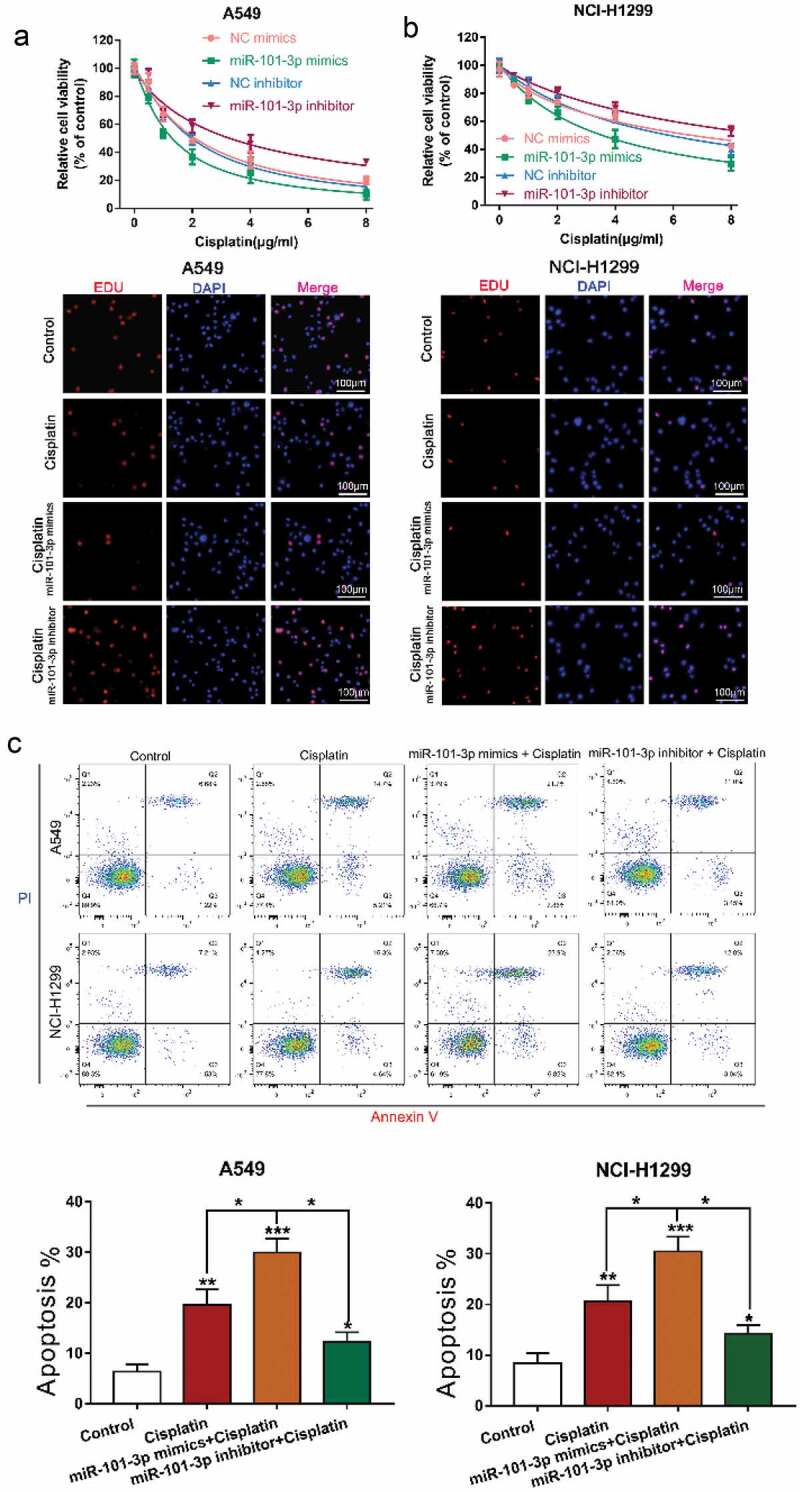
MiR-101-3p mimics increased sensitivity to cisplatin and promoted cell apoptosis

To further reveal the effect of miR-101-3p in NSCLC cells, we transfected with miR-101-3p mimics or inhibitor under cisplatin treatment and used CCK-8 assay, EdU and flow cytometry to examine cell viability, proliferation and apoptosis, respectively, the results showed that overexpression of miR-101-3p by miR-101-3p mimics increased cell viability and proliferation, however, miR-101-3p inhibitor had an opposite effect. The above results indicate that up-regulation or down-regulation of miR-101-3p could regulate cisplatin sensitivity in NSCLC cells (Figure 2 A-B). Almost immediately, we found that up-regulation of miR-101-3p enhanced the number of apoptosis cells, while miR-101-3p inhibitor reduced it (Figure 2c). This is enough to demonstrate that miR-101-3p could regulate sensitivity to cisplatin by regulating cell viability and apoptosis.

**A-B**. CCK-8 and EdU assay analyzing cell viability and proliferation after being transfected with miR-101-3p mimics or inhibitor, followed by treatment with cisplatin. **C**. Flow cytometry determined cell apoptosis following miR-101-3p mimics or inhibitor transfection, followed by treatment with cisplatin. *P < 0.05, **P < 0.01, ***P < 0.001 vs. Control.

### MiR-101-3p could interact with ATG4D expression

**Figure 3.**
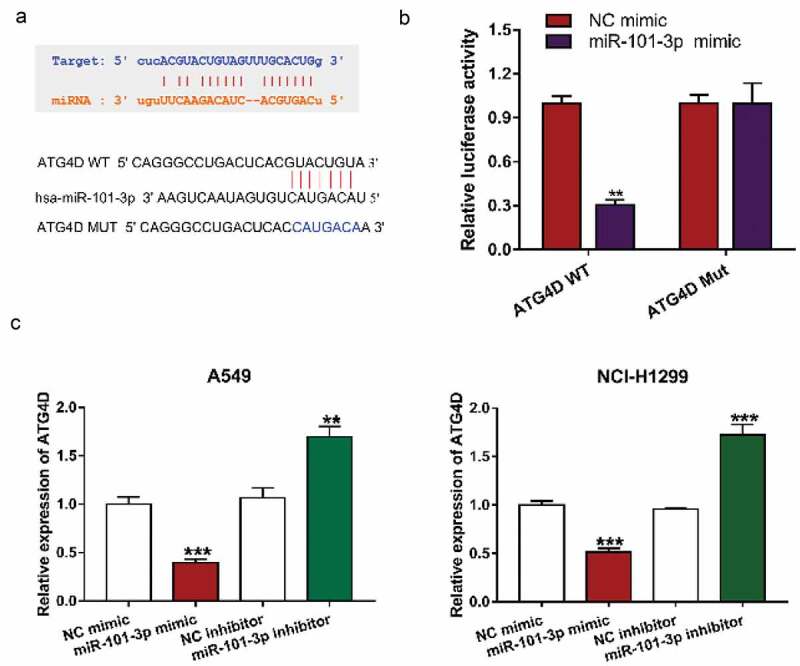
MiR-101-3p could interact with ATG4D mRNA

To elucidate the molecular mechanisms of miR-101-3p mediated autophagy, StarBase 3 (http://starbase.sysu.edu.cn/index.php) and TargetScan (http://www.targetscan.org/vert_71/) were used to analyze the expression of miRNA in NSCLC tissues and normal tissue or predicted the potential target genes of miRNA, and then found that ATG4D was a target gene for miR-101-3p (Figure 3a). Furthermore, ATG4D, as an autophagy-related gene, could regulate the autophagic flow at the autophagosome stage before fusion with lysosomes [25]. The results of Luciferase reporter assay showed that transfection with miR-101-3p mimic could down-regulate ATG4D WT luciferase activity, while it has no significant difference in ATG4D Mut between the two groups, which further verified that miR-101-3p could regulate ATG4D expression (Figure 3b). Almost immediately, we transfected with a miR-101a-3p mimic and inhibitor into NSCLC cells and determined the level of ATG4D, showing that the expression level of ATG4D was significantly downregulated by miR-101a-3p mimic treatment, whereas inhibition of miR-101a-3p upregulated ATG4D expression in NSCLC cells (Figure 3c).

A. StarBase prediction the relationship between miR-101-3p and ATG4D. B. Relative luciferase activity was determined by Dual luciferase reporter assay. **P < 0.01, vs. NC mimics. C. The expression of ATG4D was determined by qRT-PCR. **P < 0.01, vs. NC inhibitor, ***P < 0.001 vs. NC mimics (A549); ***P < 0.001 vs. NC mimics or NC inhibitor (NCI-H1299).

### The sensitivity effect of miR-101-3p was related with autophagy and regulation of ATG4D

**Figure 4.**
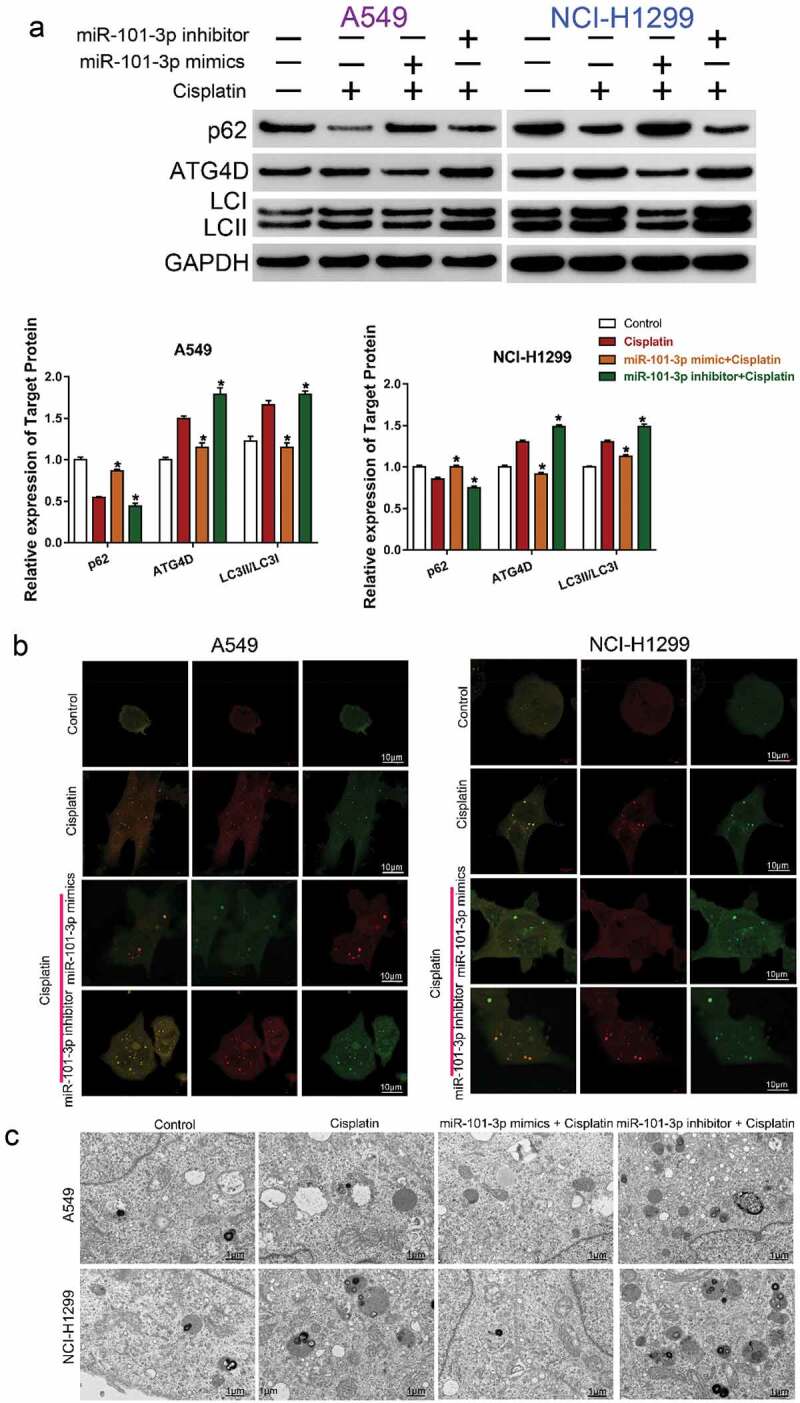
The sensitivity effect of miR-101-3p was related with autophagy and regulation of ATG4D

To explore whether miR-101-3p enhanced cisplatin sensitivity by regulating autophagy and ATG4D, the expression of autophagy-related protein ATG4D, p62 and LC3I/LC3II in different treatment group (Control, Cisplatin, miR-101-3p mimic+ Cisplatin, miR-101-3p inhibitor + Cisplatin) were detected by Western blot analysis. Transfection with miR-101a-3p mimic could inhibit cisplatin-induced the up-regulation of ATG4D expression and the increase of LC3II/LC3I ratio, the down-regulation of p62 expression, but miR-101a-3p inhibitor had the opposite effect (Figure 4 A). The expression of autophagosomes (mRFP + GFP) and autolysosomes (mRFP) represented autophagy level. Confocal microscopy analysis showed that co-treatment with miR-101-3p mimic and cisplatin could reduce the expression of autophagy in NSCLC cells compared with cisplatin treatment (Figure 4b). TEM analysis determining the features of autophagy ultrastructure indicated that cisplatin treatment increased the number of autophagosome, whereas over-expression of miR-101-3p could reduce the number of autophagosome in NSCLC cells (Figure 4c). Furthermore, we used the autophagy inducer RAPA and autophagy inhibitor 3-MA to further demonstrate the effect of miR-101-3p on enhancing cisplatin sensitivity of NSCLC cells by regulating autophagy. The results indicated that 3-MA treatment enhanced cell viability, while miR-101-3p mimics combined with 3-MA, the effect of increasing the sensitivity to cisplatin disappeared; RAPA treatment decreased cell viability, and compared with RAPA group, the effect of sensitivity to cisplatin had no significant difference in RAPA+miR-101-3p inhibitor together (Supplementary Figure 1a–B).

A. The autophagy-related protein was examined by Western Blot. *P < 0.05 vs. Cisplatin. B. The level of autophagy was examined by confocal microscopy analysis. C. TEM analysis determining the features of autophagy ultrastructure.

### Knockdown of ATG4D reversed the role of miR-101-3p inhibitor on the sensitivity to cisplatin

**Figure 5.**
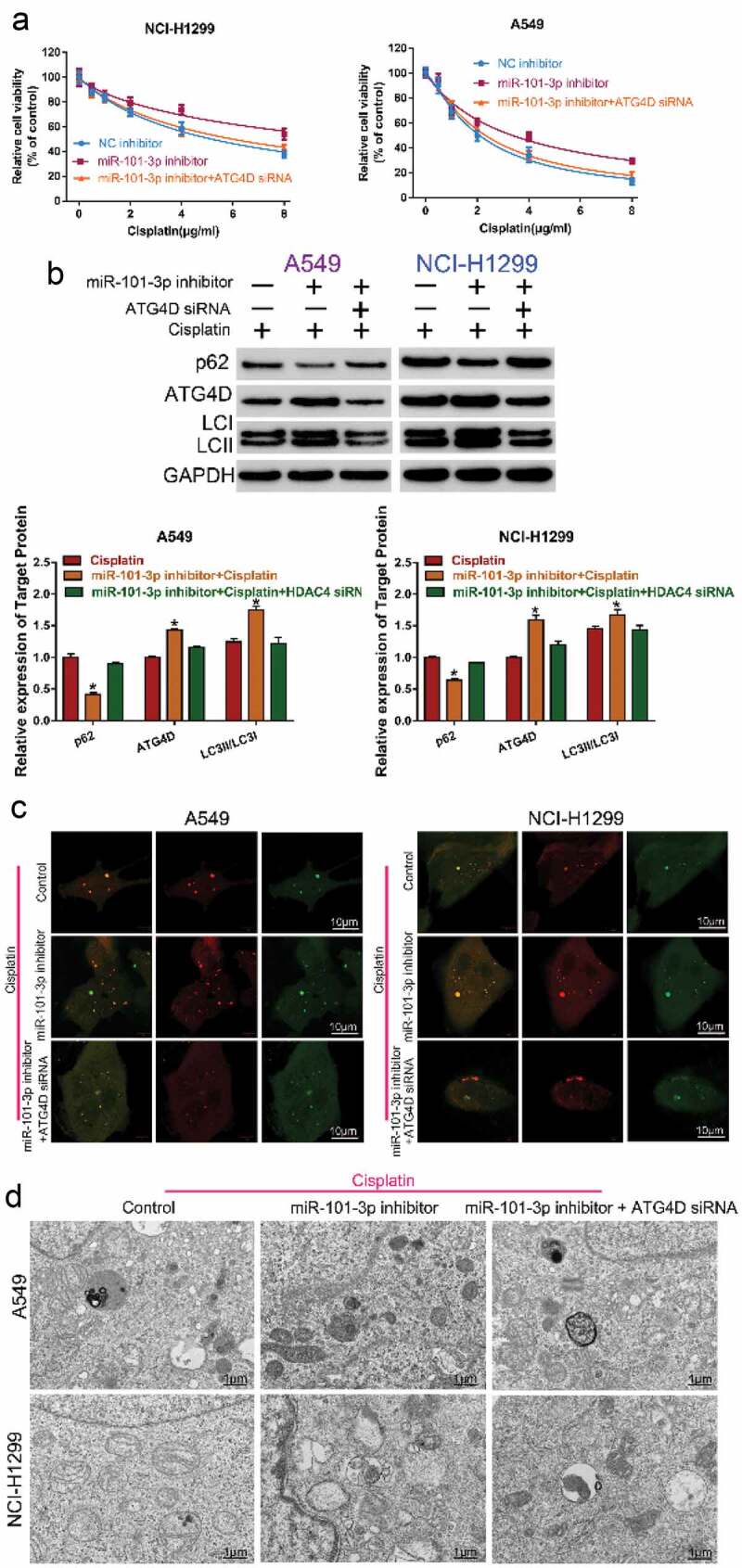
Knockdown of ATG4D reversed the role of miR-101-3p inhibitor on the sensitivity to cisplatin

To further confirm miR-101-3p regulated cisplatin sensitivity by regulation of ATG4D and autophagy, we co-transfected with miR-101-3p inhibitor and ATG4D siRNA to NSCLC cells, and compared the changes of cell activity, autophagy-related protein expression, number of autophagosomes and autolysosomes, and autophagy ultrastructure by CCK-8 assay, Western blot, confocal microscopy and TEM analysis, respectively. The results found that the miR-101-3p inhibitor treatment could decrease sensitivity to cisplatin, while knockdown of ATG4D could reverse miR-101-3p inhibitor-induced resistance to cisplatin in NSCLC cells (Figure 5 A); ATG4D siRNA also could reverse miR-101-3p inhibitor-induced the up-regulation of ATG4D and the ration of LC3II/LC3I, the down-regulation of p62 expression (Figure 5b); the total number of autophagosomes and autolysosomes was reduced after co-transfection with miR-101-3p inhibitor and ATG4D siRNA (Figure 5c); moreover, the number of autophagosome that labeled red arrow was also reduced following combined with miR-101-3p inhibitor and ATG4D siRNA (Figure 5d). These data indicated that ATG4D could reverse the role of miR-101-3p inhibitor on the sensitivity to cisplatin by autophagy.

A. The sensitivity to cisplatin transfected by miR-101-3p inhibitor, ATG4D siRNA or miR-101-3p inhibitor plus ATG4D siRNA under cisplatin treatment was detected by CCK-8 assay. B. The expression of autophagy-related protein LC3II/LC3I, p62 and ATG4D was examined by Western Blot. *P < 0.05 vs. Control. C. The level of autophagy after co-transfection with miR-101-3p inhibitor and ATG4D siRNA were examined by confocal microscopy analysis. D. TEM analysis determining the features of autophagy ultrastructure.

## Up-regulated miR-101-3p could inhibit tumor the effect of transplanted tumor growth in vivo

**Figure 6.**
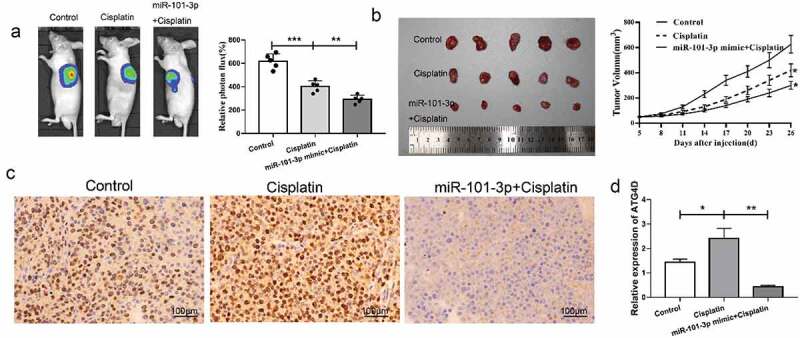
MiR-101-3p mimics could inhibit tumor the effect of transplanted tumor growth in vivo

Next, we further verify the effect of miR-101-3p in vivo, in vivo imaging showed that compared with control, the tumor volume decreased after cisplatin treatment, and compared to the cisplatin group, the tumor volume reduced following transfection with miR-101-3p mimic, the difference was statistically significant (Figure 6a). MiR-101-3p up-regulation plus cisplatin could obviously inhibit tumor grown, which had a similar effect on in vitro experiments (Figure 6b). Immunohistochemistry analysis showed that the cisplatin group had obvious brown positively expressed cells, and the level of ATG4D was reduced in the cisplatin group, while it was decreased following combined with miR-101-3p agomiR (Figure 6c). The above data revealed that miR-101-3p was up-regulated to inhibit ATG4D expression in the transplanted tumor tissues.

**A-B**. The mice vivo imaging and the size of tumor detection analysis. *P < 0.05 vs. Control; **P < 0.01, ***P < 0.001. **C-D**. Immunohistochemistry analysis of the expression of ATG4D in different treated groups. *P < 0.05, **P < 0.01.

## Discussion

Cisplatin is one of the best and first chemotherapeutic drugs for the treatment of NSCLC [26]; however, cisplatin does not show its highest potential because of side effects and drug resistance. Therefore, an understanding of the mechanisms of cisplatin resistance will be important to improve the efficacy of cisplatin treatment. It is well known that miRNAs are associated with drug resistance in various cancer cells [27,28]. Treatment with miR-423 inhibitor could significantly enhance sensitivity to drugs in breast cancer by reducing cell viability and increasing the apoptotic cells. We used TCGA and GEO databases to verify some miRNAs that are associated with cisplatin sensitivity in NSCLC (number: GSE135918). We screened top 15 differential expression miRNAs, identified that only miR-101-3p was significantly downregulated in 5 paired primary LUAD tissues and had a significant difference in survival curve. It was also down-regulated in NSCLC cancer cells, which suggested that miR-101-3p may be more suitable for the research objective of this article.

Autophagy is a conservative metabolic process, which is induced quickly to ensure the survival of tumor cells facing a harsh tumor microenvironment, for instance, hypoxia, starvation or chemotherapy drugs. Autophagy is also associated with the tumor development and progression, such as cell viability, cell resistance, and cell metastasis [29–31]. It has been reported that regulation of autophagy may be as an effective treatment strategy to improve the efficacy of many antitumor drugs [32–34]. Recent studies also revealed that miRNAs could regulate drug treatment-induced autophagy in many different cancers [27,28]. For instance, transfection with miR-874 mimics could sensitize gastric cancer cells to chemotherapy through regulating ATG16L1 and inhibiting autophagy [35]. Transfection with miR-153-3p mimics also could increase sensitivity to gefitinib in NSCLS cells via inhibiting autophagy and ATG5 [36]. Based on gene database and StarBase software analysis, we identified that the suitable miRNA (miR-101-3p) which was down-regulated in LUAD tissue and NSCLC cells. Later, it was also found that miR-101-3p could decrease the regulation of cisplatin induced autophagy in NSCLC cells. The activity of autophagy was associated with the number of LC3-II or the rate of LC3-II/LC3-I [37,38]. P62 can select the degradation of autophagy by combining cargo with autophagy mechanism, which was also one of the most famous autophagy receptors [39]. The present study has demonstrated that transfection with miR-101a-3p and treatment with cisplatin could inhibit LC3II/LC3I ratio, the expression of autophagosomes and autolysosomes, increase p62 expression in NSCLC cells, but miR-101a-3p inhibitor had the opposite.

Autophagy-related protein 4D (ATG4D) and mTOR have been identified to play an important effect in the formation of autophagosome [40]. Up-regulation of miR-101 could enhance cisplatin-induced apoptosis and sensitivity to cisplatin in HCC cells by inhibiting autophagy [41]. However, the understanding of the mechanisms of miR-101-3p on resistance to cisplatin in NSCLC cells was not further researched. This study identified that ATG4D was a target gene for miR-101-3p in NSCLC cells and inhibition of ATG4D reversed the role of miR-101-3p inhibitor on the sensitivity to cisplatin in NSCLC cells. It also demonstrated that transfected with ATG4D siRNA also could reverse miR-101-3p inhibitor-induced the up-regulation level of autophagy. However, we also proved that miR-101-3p mimic could enhance the sensitivity to cisplatin in vivo. Additionally, further identification of the precise mechanisms underlying of ATG4D is needed, which will be the direction and focus of our future research.

## In conclusion

Taken together, our findings demonstrated that over-repression of miR-101-3p enhanced cisplatin sensitivity by inhibiting cell viability and increasing cell apoptosis, inducing autophagy in NSCLC cells, and the potential mechanism was related with regulating of ATG4D expression, suggesting that the miR-101a-3p-ATG4D axis may become a novel prognostic and therapeutic target for overcoming cisplatin resistance in NSCLC.

## Supplementary Material

Supplemental Material

## Data Availability

The datasets used and/or analyzed during the current study are available from the corresponding author upon reasonable request.

## References

[1] Hirsch FR, Scagliotti GV, Mulshine JL, et al. Lung cancer: current therapies and new targeted treatments. Lancet. 2017;389:299–311. 27574741 10.1016/S0140-6736(16)30958-8

[2] Bao Y, Zhang Y, Lu Y, et al. Overexpression of microRNA-9 enhances cisplatin sensitivity in hepatocellular carcinoma by regulating EIF5A2-mediated epithelial-mesenchymal transition. Int J Biol Sci. 2020;16:827–837. 32071552 10.7150/ijbs.32460 PMC7019138

[3] Hill DP, Harper A, Malcolm J, et al. Cisplatin-resistant triple-negative breast cancer subtypes: multiple mechanisms of resistance. BMC Cancer. 2019;19:1039. 31684899 10.1186/s12885-019-6278-9 PMC6829976

[4] Liu P, Li X, Cui Y, et al. LncRNA-MALAT1 mediates cisplatin resistance via miR-101-3p/VEGF-C pathway in bladder cancer. Acta Biochim Biophys Sin (Shanghai). 2019;51:1148–1157. 31650173 10.1093/abbs/gmz112

[5] Zhou F, Yang X, Zhao H, et al. Down-regulation of OGT promotes cisplatin resistance by inducing autophagy in ovarian cancer. Theranostics. 2018;8:5200–5212. 30555541 10.7150/thno.27806 PMC6276088

[6] Gong T, Cui L, Wang H, et al. Knockdown of KLF5 suppresses hypoxia-induced resistance to cisplatin in NSCLC cells by regulating HIF-1alpha-dependent glycolysis through inactivation of the PI3K/Akt/mTOR pathway. J Transl Med. 2018;16:164. 29898734 10.1186/s12967-018-1543-2 PMC6000925

[7] Zhou Q, Huang SX, Zhang F, et al. MicroRNAs: a novel potential biomarker for diagnosis and therapy in patients with non-small cell lung cancer. Cell Prolif. 2017;50(6):e12394. 10.1111/cpr.12394 PMC6529072 28990243

[8] Ma X, Feng J, Lu M, et al. microRNA-501-5p promotes cell proliferation and migration in gastric cancer by downregulating LPAR1. J Cell Biochem. 2020;121:1911–1922. 31746031 10.1002/jcb.29426

[9] Zhao M, Chen N, Li X, et al. MiR-19a modulates hypoxia-mediated cell proliferation and migration via repressing PTEN in human pulmonary arterial smooth muscle. Life Sci. 2019;239:116928. 31682848 10.1016/j.lfs.2019.116928

[10] Baumgartner U, Berger F, Hashemi Gheinani A, et al. miR-19b enhances proliferation and apoptosis resistance via the EGFR signaling pathway by targeting PP2A and BIM in non-small cell lung cancer. Mol Cancer. 2018;17:44. 29455644 10.1186/s12943-018-0781-5 PMC5817797

[11] Jiang J, Xie C, Liu Y, et al. Up-regulation of miR-383-5p suppresses proliferation and enhances chemosensitivity in ovarian cancer cells by targeting TRIM27. Biomed Pharmacother. 2019;109:595–601. 30399596 10.1016/j.biopha.2018.10.148

[12] Caporali S, Amaro A, Levati L, et al. miR-126-3p down-regulation contributes to dabrafenib acquired resistance in melanoma by up-regulating ADAM9 and VEGF-A. J Exp Clin Cancer Res. 2019;38:272. 31227006 10.1186/s13046-019-1238-4 PMC6588909

[13] Liao J, Lin J, Lin D, et al. Down-regulation of miR-214 reverses erlotinib resistance in non-small-cell lung cancer through up-regulating LHX6 expression. Sci Rep. 2017;7:781. 28396596 10.1038/s41598-017-00901-6 PMC5429707

[14] Sun W, Zhang Q, Wu Z, et al. miR-101-3p sensitizes hepatocellular carcinoma cells to oxaliplatin by inhibiting Beclin-1-mediated autophagy. Int J Clin Exp Pathol. 2019;12:2056–2065. 31934027 PMC6949619

[15] Li L, Shao MY, Zou SC, et al. MiR-101-3p inhibits EMT to attenuate proliferation and metastasis in glioblastoma by targeting TRIM44. J Neurooncol. 2019;141:19–30. 30539341 10.1007/s11060-018-2973-7

[16] Zhang X, He X, Liu Y, et al. MiR-101-3p inhibits the growth and metastasis of non-small cell lung cancer through blocking PI3K/AKT signal pathway by targeting MALAT-1. Biomed Pharmacother. 2017;93:1065–1073. 28738500 10.1016/j.biopha.2017.07.005

[17] Li B, Xie D, Zhang H. MicroRNA-101-3p advances cisplatin sensitivity in bladder urothelial carcinoma through targeted silencing EZH2. J Cancer. 2019;10:2628–2634. 31258770 10.7150/jca.33117 PMC6584933

[18] Wang C, Liu B. miR-101-3p induces autophagy in endometrial carcinoma cells by targeting EZH2. Arch Gynecol Obstet. 2018;297:1539–1548. 29691644 10.1007/s00404-018-4768-7

[19] Onorati AV, Dyczynski M, Ojha R, et al. Targeting autophagy in cancer. Cancer. 2018;124:3307–3318. 29671878 10.1002/cncr.31335 PMC6108917

[20] Li X, He S, Ma B. Autophagy and autophagy-related proteins in cancer. Mol Cancer. 2020;19:12. 31969156 10.1186/s12943-020-1138-4 PMC6975070

[21] Smith AG, Macleod KF. Autophagy, cancer stem cells and drug resistance. J Pathol. 2019;247:708–718. 30570140 10.1002/path.5222 PMC6668344

[22] Meng CY, Zhao ZQ, Bai R, et al. MicroRNA22 regulates autophagy and apoptosis in cisplatin resistance of osteosarcoma. Mol Med Rep. 2020;22:3911–3921. 33000186 10.3892/mmr.2020.11447 PMC7533487

[23] Yu JL, MicroRNA GX. 1301 inhibits cisplatin resistance in human ovarian cancer cells by regulating EMT and autophagy. Eur Rev Med Pharmacol Sci. 2020;24:1688–1696. 32141535 10.26355/eurrev_202002_20343

[24] Liang L, Peng Y, Zhang J, et al. Deubiquitylase USP7 regulates human terminal erythroid differentiation by stabilizing GATA1. Haematologica. 2019;104:2178–2187. 30872372 10.3324/haematol.2018.206227 PMC6821630

[25] Del Bello B, Marcolongo P, Ciarmela P, et al. Autophagy up-regulation by ulipristal acetate as a novel target mechanism in the treatment of uterine leiomyoma: an in vitro study. Fertil Steril. 2019;112:1150–1159. 31843092 10.1016/j.fertnstert.2019.08.007

[26] Griesinger F, EE K, Kayaniyil S, et al. Efficacy and safety of first-line carboplatin-versus cisplatin-based chemotherapy for non-small cell lung cancer: a meta-analysis. Lung Cancer. 2019;135:196–204. 31446995 10.1016/j.lungcan.2019.07.010

[27] Wang ZC, Huang FZ, Xu HB, et al. MicroRNA-137 inhibits autophagy and chemosensitizes pancreatic cancer cells by targeting ATG5. Int J Biochem Cell Biol. 2019;111:63–71. 30710750 10.1016/j.biocel.2019.01.020

[28] Li H, Chen L, Li JJ, et al. miR-519a enhances chemosensitivity and promotes autophagy in glioblastoma by targeting STAT3/Bcl2 signaling pathway. J Hematol Oncol. 2018;11:70. 29843746 10.1186/s13045-018-0618-0 PMC5975545

[29] Soni M, Patel Y, Markoutsa E, et al. Autophagy, cell viability, and chemoresistance are regulated by miR-489 in Breast cancer. Mol Cancer Res. 2018;16:1348–1360. 29784669 10.1158/1541-7786.MCR-17-0634 PMC6843115

[30] Li YJ, Lei YH, Yao N, et al. Autophagy and multidrug resistance in cancer. Chin J Cancer. 2017;36:52. 28646911 10.1186/s40880-017-0219-2 PMC5482965

[31] Mowers EE, Sharifi MN, Macleod KF. Autophagy in cancer metastasis. Oncogene. 2017;36:1619–1630. 27593926 10.1038/onc.2016.333 PMC5337449

[32] Heqing Y, Bin L, Xuemei Y, et al. The role and mechanism of autophagy in sorafenib targeted cancer therapy. Crit Rev Oncol Hematol. 2016;100:137–140. 26920575 10.1016/j.critrevonc.2016.02.006

[33] Pan X, Chen Y, Shen Y, et al. Knockdown of TRIM65 inhibits autophagy and cisplatin resistance in A549/DDP cells by regulating miR-138-5p/ATG7. Cell Death Dis. 2019;10:429. 31160576 10.1038/s41419-019-1660-8 PMC6546683

[34] Chen C, Lu L, Yan S, et al. Autophagy and doxorubicin resistance in cancer. Anticancer Drugs. 2018;29:1–9. 29099416 10.1097/CAD.0000000000000572

[35] Huang H, Tang J, Zhang L, et al. miR-874 regulates multiple-drug resistance in gastric cancer by targeting ATG16L1. Int J Oncol. 2018;53:2769–2779. 30320370 10.3892/ijo.2018.4593

[36] Zhang W, Dong YZ, Du X, et al. MiRNA-153-3p promotes gefitinib-sensitivity in non-small cell lung cancer by inhibiting ATG5 expression and autophagy. Eur Rev Med Pharmacol Sci. 2019;23:2444–2452. 30964170 10.26355/eurrev_201903_17391

[37] Tanida I, Ueno T, Kominami E. LC3 and autophagy. Methods Mol Biol. 2008;445:77–88. 18425443 10.1007/978-1-59745-157-4_4

[38] Wang J, Liu Y, Li XH, et al. Curcumin protects neuronal cells against status-epilepticus-induced hippocampal damage through induction of autophagy and inhibition of necroptosis. Can J Physiol Pharmacol. 2017;95:501–509. 28177687 10.1139/cjpp-2016-0154

[39] Mijaljica D, Nazarko TY, Brumell JH, et al. Receptor protein complexes are in control of autophagy. Autophagy. 2012;8:1701–1705. 22874568 10.4161/auto.21332 PMC3494607

[40] Zhang X, Zhao P, Wang C, et al. SNHG14 enhances gemcitabine resistance by sponging miR-101 to stimulate cell autophagy in pancreatic cancer. Biochem Biophys Res Commun. 2019;510:508–514. 30737032 10.1016/j.bbrc.2019.01.109

[41] Xu Y, An Y, Wang Y, et al. miR-101 inhibits autophagy and enhances cisplatin-induced apoptosis in hepatocellular carcinoma cells. Oncol Rep. 2013;29:2019–2024. 23483142 10.3892/or.2013.2338

