# Supplementary material for: Up-regulation of microRNA miR-101-3p enhances sensitivity to cisplatin via regulation of small interfering RNA (siRNA) Anti-human AGT4D and autophagy in non-small-cell lung carcinoma (NSCLC)
Source: Bioengineered. 2021 Oct 25;12(1):8435–46. doi: 10.1080/21655979.2021.1982274 (PMC8806688; doi:10.1080/21655979.2021.1982274)
Supplement: Supplemental Material [file KBIE_A_1982274_SM9246.docx]

**Supplementary files**


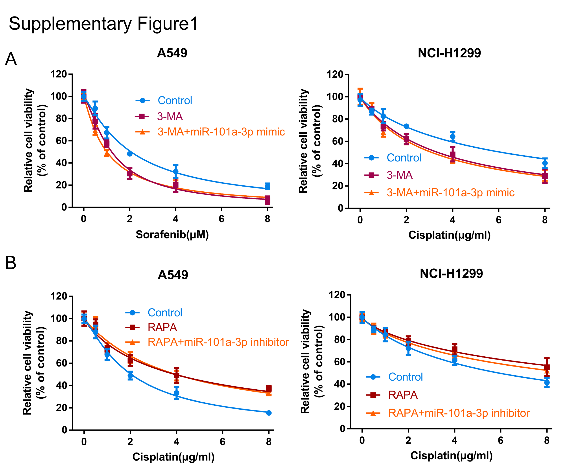


**Supplementary Figure 1A−B**

A. CCK-8 assay determined the cell viability following treatment with 3-MA or combined with miR-101-3 mimic, which was exposed to cisplatin in NSCLC cells. B. NSCLC cells was treated with RAPA or plus with miR-101-3p inhibitor, followed with cisplatin by CCK-8 analysis.
